# Proteomic Analysis of the Metabolic Response of UVA-Exposed Melanocytes Following Co-Treatment with Cannabigerol and 3-*O*-Ethylascorbic Acid

**DOI:** 10.3390/cells15110965

**Published:** 2026-05-23

**Authors:** Magda Mucha, Alena Ryšavá, Iwona Jarocka-Karpowicz, Audrius Maruška, Elżbieta Skrzydlewska, Agnieszka Gęgotek

**Affiliations:** 1 Department of Analytical Chemistry, Medical University of Bialystok, Kilinskiego 1, 15-069 Bialystok, Poland; mmucha1106@gmail.com (M.M.); iwona.jarocka-karpowicz@umb.edu.pl (I.J.-K.); elzbieta.skrzydlewska@umb.edu.pl (E.S.); 2 Department of Medical Chemistry and Biochemistry, Faculty of Medicine and Dentistry, Palacký University, Hněvotínská 3, 779 00 Olomouc, Czech Republic; rysava.alena@hotmail.com; 3 Instrumental Analysis Open Access Center, Faculty of Natural Sciences, Vytautas Magnus University, Vileikos 8, LT-44404 Kaunas, Lithuania; audrius.maruska@vdu.lt

**Keywords:** melanocytes, UVA radiation, cannabigerol, 3-*O*-ethyl ascorbic acid, proteomics, skin protection

## Abstract

The aim of this study was to analyze the effect of concomitant use of cannabigerol (CBG) and 3-*O*-ethylascorbic acid (EAA) on changes in the proteome of UVA-irradiated skin melanocytes, with particular emphasis on adduct formation between lipid peroxidation products and metabolically important proteins. Proteomic analysis allowed the identification of 1248 proteins with statistically significantly changed expression following melanocytes irradiation and/or incubation with CBG/EAA. The top 25 proteins with the most strongly differentially abundant expression included proteins involved in cell protection/antioxidant response, as well as pro-inflammatory and proapoptotic signalization. Moreover, in melanocytes irradiated with UVA, the levels of lipid peroxidation product, 4-hydroxynonenal (4-HNE) and its protein adducts were increased, as well as significant changes in the profile of proteins modified by 4-HNE were observed. CBG and EAA, especially when used together, largely reverse these effects. This study for the first time demonstrated the combined effect of CBG and EAA on the proteome of melanocytes after their exposure to UVA radiation, which applies to both changes in protein expression and intracellular signaling based on proteins modified by 4-HNE. It can be suggested that CBG and EAA may provide melanocytes with effective protection against the effects of oxidative stress and perhaps even protect the skin from carcinogenesis.

## 1. Introduction

Ultraviolet A (UVA) radiation (320–400 nm) plays a key role in both the physiology and pathology of human skin cells, including pigment cells—melanocytes [1,2], leading to increased generation of reactive oxygen species (ROS), which in melanocytes is further enhanced by melanin-mediated photosensitization [1], the biosynthesis of which is also associated with the generation of ROS, including hydrogen peroxide (H_2_O_2_) and singlet oxygen (^1^O_2_) [3]. In contrast, ROS can directly stimulate melanocyte proliferation or, in extreme cases, induce DNA modifications with oxidative changes that induce melanomagenesis [4]. Additionally, peroxynitrite, formed from superoxide anion (O_2_^−^) and nitric oxide (NO), can solubilize melanin, enabling it to directly interact with nuclear DNA and enhancing genomic instability of melanocytes [5].

Melanocytes, due to their location in the epidermis, are exceptionally sensitive to the pro-oxidant effects of UVA radiation and oxidative stress resulting from melanin biosynthesis [6,7]. Consequently, these cells accumulate oxidative changes at higher levels than neighboring keratinocytes [6]. Moreover, the CPD to oxidative changes ratio is significantly lower in melanocytes (~1.4) than in keratinocytes (~5.2), indicating greater oxidative changes in melanocytes [6]. Furthermore, UVA radiation in melanocytes also induces the accumulation of p53 protein, activating DNA damage response pathways, including apoptosis [8]. Regardless of the above changes, overproduction of ROS following melanocyte exposure to UVA radiation enhances the generation of free radicals and enzymatic lipid metabolites involved in pro-oxidant cell signaling [9], which ultimately promotes the formation of adducts, including lipid peroxidation products (e.g., 4-hydroxynonenal; 4-HNE and malondialdehyde; MDA), with proteins [10,11]. These modifications lead to changes in the structure and conformation of proteins, which induce disturbances in their activity and often result in changes in intracellular signaling regulating antioxidant, inflammatory, or apoptotic cellular responses [12].

Given the pro-oxidant effects of UVA radiation, antioxidants are sought, including both plant-derived compounds, such as phytocannabinoids (including cannabigerol—CBG), and synthesized vitamin derivatives, including 3-*O*-ethylascorbic acid (EAA). CBG is known to significantly reduce ROS generation while simultaneously increasing superoxide dismutase (SOD) activity in skin cells, which promotes the reduction of lipid peroxidation and inflammation by inhibiting the cyclooxygenase-1/2 and lipoxygenase-5 (COX-1/2 and LOX-5) pathways [13,14,15]. EAA, a stable derivative of ascorbic acid, enhances antioxidant defense, including through the cytoprotective factor Nrf2 [16]. Its lipophilic nature, on the other hand, enables the protection of lipid membrane components with a reduction in markers of lipid peroxidation (MDA and 4-HNE) [13]. Consequently, the combined use of CBG and EAA, by protecting membrane lipids and also silencing pro-inflammatory signaling, demonstrates synergy in protecting skin cells from UVA-induced oxidative stress, as previously observed in the case of keratinocytes [13,17].

However, there is currently no knowledge about the effect of combined CBG and EAA use on melanocytes’ metabolism and function, nor whether these compounds will synergize in protecting/regenerating these cells after exposure to UVA radiation, which is particularly important, as metabolic modifications of melanocytes under the influence of UVA radiation may lead to their neoplastic transformation into melanoma [18].

Therefore, this study aims to assess the effect of simultaneous use of CBG and EAA on changes in the melanocyte proteome induced by UVA radiation, with particular emphasis on adduct formation between lipid peroxidation products and metabolically important proteins that lead to disturbances in their function and, consequently, cellular metabolism.

## 2. Materials and Methods

### 2.1. Cell Culture and Treatment

Human primary epidermal melanocytes (ATCC PCS-200-012) isolated from the foreskin of a newborn were obtained from the American Type Culture Collection ATCC^®^ (Manassas, VA, USA). The cell line was validated by STR profiling and tested negative for mycoplasma. Cells were cultured according to the manufacturer’s guidelines in a humid atmosphere of 5% CO_2_ and a temperature of 37 °C, using Dermal Cell Basal Medium (DCBM, ATCC-PCS-200-030) supplemented with Melanocyte Growth Kit (ATCC-PCS-200-041) and antibiotics: 50 U/mL penicillin and 50 µg/mL streptomycin. Melanocytes from passage 3 were used in the experiment.

After reaching 90% confluency, cells were exposed to UVA radiation with a total dose 18 J/cm^2^ (365 nm; intensity of 4.08 mW/cm^2^; Bio-Link Crosslinker BLX 365; Vilber Lourmat, Germany). The radiation dose was chosen in reference to approximately 70% of cell viability as measured by the MTT (3-(4,5-dimethylthiazol-2-yl)-2,5-diphenyltetrazolium bromide) method [19]. To examine the effect of CBG and EAA, melanocytes were incubated for 24 h in a medium supplemented with CBG (1 µM; diluted in ethanol; final ethanol concentration in medium was 0.3%) and/or EAA (150 µM, diluted in phosphate-buffered saline, PBS). Independently, untreated and unirradiated cells were cultured as a control group.

**Figure 1:**
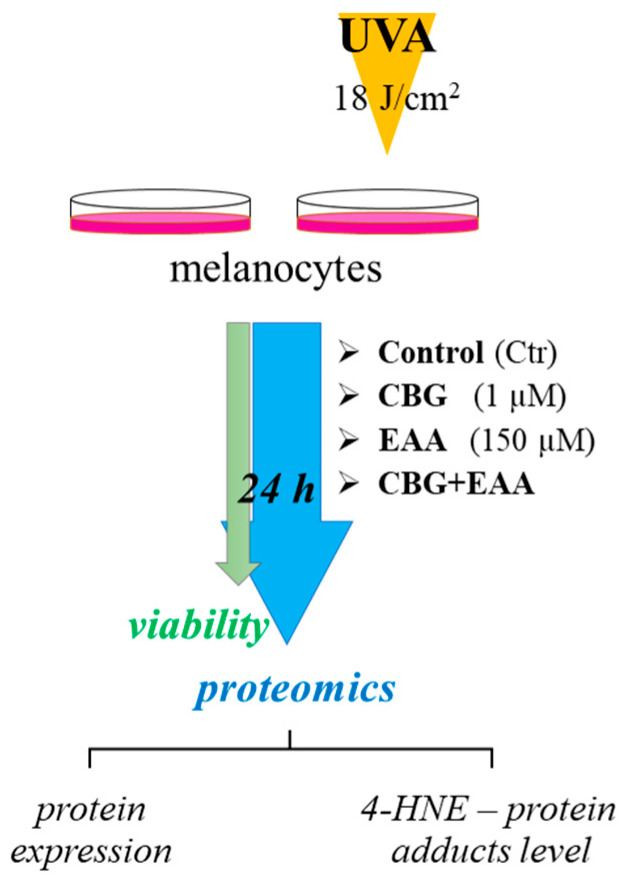
Scheme of the experiment conducted on UVA (18 J/cm^2^) irradiated melanocytes treated for 24 h with cannabigerol (CBG, 1 µM) or/and 3-O-ethyl ascorbic acid (EAA, 150 µM) in vitro in 100 mm plastic adherent culture plate.

To eliminate the ethanol’s differentiating effect on the cells, all groups were cultured in medium containing 0.3% of ethanol, which does not show cytotoxicity in in vitro cell cultures [20]. After 24 h incubation, a viability test and proteomic analyses were performed. The scheme of the experiment is presented in Figure 1.

### 2.2. Cells Viability Measurement

For cell viability measurement, melanocytes were seeded and treated in 96-well plates. Cell seeding density was 10^4^ cells per well. Non-irradiated melanocytes, as well as cells following their exposure to UVA radiation (18 J/cm^2^), were incubated for 24 h in a medium supplemented with CBG (0.5–15 µM; obtained after appropriate dilution from a 32 mM solution of CBG in ethanol; final ethanol concentration in medium was 0.3%) and/or EAA (50–250 µM, diluted in phosphate-buffered saline, PBS). To prevent solvent-induced effects, all cell media contained the same amount of PBS and ethanol. Measurements for each experimental group were performed in 5 independent wells. Following 24 h incubation, medium from each well was removed, and cells were incubated with 0.25 mg/mL MTT solution in PBS for 40 min at a temperature of 37 °C (conditions optimized for melanocytes). MTT was removed, and cells were lysed using dimethyl sulfoxide. The absorbance of formed formazan was read at 570 nm [19] using a Multiskan GO Microplate Spectrophotometer (Thermo Scientific, USA). Background absorbance was corrected by subtracting the absorbance of blank wells (wells that contain only culture medium, without seeded cells, incubated with MTT like the other wells) from all experimental well readings. Results were presented as a percentage of the value obtained for control cells.

### 2.3. Evaluation of 4-Hydroxy-2-Nonenal (4-HNE) Level

The level of 4-HNE was determined using the GC-MS/MS method [21]. 4-HNE was converted into its O-PFB-oxime-TMS derivative via derivatization with O-(2,3,4,5,6-pentafluorobenzyl) 0.05 mol/L hydroxyamine hydrochloride. Samples were then extracted with hexane and, after evaporation under argon, suspended in N,O-bis(trimethylsilyl)trifluoroacetamide. The samples were subsequently analyzed using a 7890A GC–7000 quadruple MS/MS (Agilent Technologies, Palo Alto, CA, USA) in a selected ion monitoring (SIM) mode: *m*/*z*; 352.0, 271.2 (4-HHE-PFB-TMS) and *m*/*z*; 307.0 (internal standard derivatives). Results were presented as percentage changes of 4-HNE in comparison to the control group.

### 2.4. Proteomic Analysis

Approximately 2 × 10^6^ cells per plate following UVA irradiation and/or treatment with CBG/EAA were scraped into PBS (10 °C) and disintegrated by sonication on ice. Following centrifugation, the total protein content of the cell lysate was measured using the Bradford assay [22]. MS-based proteomic analysis was performed following electrophoretic pre-separation of proteins and their in-gel digestion, in order to remove the non-protein biological matrix of the samples and to separate a complex protein mixture into less complex fractions. The volume of lysates containing 30 µg was remixed with sample loading buffer (Laemmle buffer containing 5% 2-mercaptoethanol), heated at 95 °C for 7 min, and electrophoretically (100 V) separated on 10% SDS-PAGE gels. Gels were fixed for 1 h with 40% methanol and 10% acetic acid and then stained for 4 h with Coomassie brilliant blue. Gels were destained with 25% methanol and rinsed with milli Q water. Complete sample lanes were cut out. Gel parts were destaining based on gel dehydration/rehydration using alternating addition of ammonium bicarbonate buffer (AMBIC, 25 mM, for 10 min) and acetonitrile (for 25 min) in room temperature. Next, proteins in gel parts were reduced with 10 mM dithiothreitol (30 min, 60 °C) and alkylated with 50 mM iodoacetamide (30 min, room temperature, in the dark). Following overnight in-gel digestion using trypsin (Promega, Madison, WI, USA), peptides were extracted from the gel by dehydration with acetonitrile. Obtained mixtures were dried under inert gas and dissolved in 5% acetonitrile (with 0.1% formic acid).

Direct mass spectrometry analysis was performed using a QExactive HF mass spectrometer with an electrospray ionization source operated using Xcalibur 4.1 (Thermo Fisher Scientific, Bremen, Germany) following preliminary peptides separation on the 150 mm × 75 µm PepMap RSLC capillary analytical C18 column (Dionex, LC Packings) using Ultimate 3000 (Dionex, Idstein, Germany) as described previously [17]. The mass spectrometer was operated in positive mode, and survey MS scans were conducted in the 200–2000 *m*/*z* range with a resolution of 120,000. In subsequent scans, the top ten most intense ions were isolated, fragmented, and analyzed at 30,000 resolution. Details of the analysis were described in a Supplementary File S1.

The raw data were processed using Proteome Discoverer 2.0 (Thermo Fisher Scientific, Bremen, Germany), and input data were searched against the UniProtKB—SwissProt database (taxonomy: Homo sapiens, release 2025-04). As a dynamic modification of the chosen amino acids (cysteine, lysine, and histidine), adduct formation with 4-HNE was set [23]. Details of the analysis were described in a Supplementary File S1. Only proteins with at least two unique peptides longer than 6 amino acid residues were taken for statistical analysis. The signal intensities of the precursor ions were used for label-free semi-quantification of proteins. The 4-HNE-modified protein semi-quantification was done based on the analysis of peak intensity of modified peptides, the quantity of which is adequate for the levels of proteins from which they come [23].

### 2.5. Statistical Analysis

For viability measurement and determination of 4-HNE level, analysis was performed in 5 independent biological and 3 analytical replicates. Results were presented as mean values with standard deviation (SD), and their statistical analysis was performed using univariate analysis of one-way (ANOVA, Fisher’s least significant differences (LSD) < 5%).

Proteomic analyses were performed in 3 independent biological and 3 analytical replicates. The results from individual protein label-free semi-quantification were subjected to data imputation (missing values were replaced by 1/5 of the minimal positive values of their corresponding variables), assuming that the missing data are not random but represent low concentrations below the detection limit. To obtain a normal distribution, the results were normalized by the median of the protein intensities, and log-transformed using open-source software MetaboAnalyst 6.0 (http://www.metaboanalyst.ca; accessed on 11 September 2025) [24,25]. The normalization view is included in the supplements (Supplementary File S2). The selected normalization method gave a distribution closest to normal; however, other statistical approaches for proteins with the largest fold change gave similar results. Using the same software for statistical analysis of protein abundance, and due to the lack of homogeneity of variance, the non-parametric Kruskal–Wallis test was applied (false discovery rate (FDR) < 5%). Also, a heatmap with protein clustering and principal component analysis (PCA) was done using MetaboAnalyst 6.0 (http://www.metaboanalyst.ca; accessed on 11 September 2025) [24]. A hierarchical clustering heatmap was created on normalized data with Euclidean distance measurement using the Ward method for clustering. For the designation of the top 25 differentially abundant proteins, the non-parametric Kruskal–Wallis test was applied as before. The sample distribution in PCA was computed using the Euclidean distance. Protein functions were determined based on functional classification using the Protein Analysis Through Evolutionary Relationships Classification System (PANTHER 17.0; PANTHER GO-Slim annotation) [26].

## 3. Results

**Figure 2:**
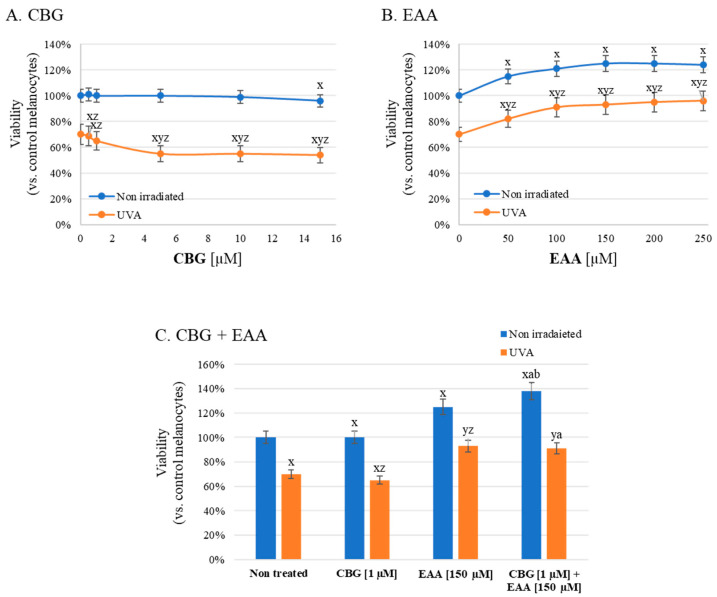
Effect of cannabigerol (CBG, 0.5–15 µM) (**A**), 3-O-ethyl ascorbic acid (EAA, 50–250 µM) (**B**), and both compounds used together (CBG (1 µM) + EAA (150 µM)) (**C**), on skin melanocytes viability cultured in vitro under standard conditions, as well as following UVA irradiation (18 J/cm^2^). Results obtained using MTT assay for 3 independent biological replications. Mean values ± SD are presented with statistically significant differences comparing to: x—control cells; y—UVA irradiated cells; z—cells not irradiated, but treated with the same concentration of CBG or EAA; a—non irradiated or UVA irradiated cells treated only with CBG (1 µM); b—non irradiated or UVA irradiated cells treated only with EAA (150 µM).

The obtained results demonstrated that CBG and EAA in the range of concentrations used, both in single application and co-application, had significant, but divergent effects on melanocyte viability, especially those exposed to UVA radiation (Figure 2). While in the case of non-irradiated melanocytes, treatment with CBG (0.5–10 µM) did not significantly affect cell viability, following melanocyte exposure to UVA, CBG additionally reduced the viability of these cells by 20%. On the other hand, EAA used alone significantly enhanced cell viability in non-irradiated, as well as UVA-irradiated melanocytes. In the case of CBG, the selected concentration (1 µM) for further analysis was the highest that did not induce cytotoxicity to unirradiated cells, as well as after their exposure to UVA radiation. For EAA, the chosen concentration (150 µM) caused the highest viability rate of unirradiated melanocytes and one of the highest for cells exposed to UVA radiation. Notably, the combined treatment with 1 µM CBG and 150 µM EAA exerted the most substantial effect. In non-irradiated melanocytes, viability increased to nearly 140%, whereas in the UVA-irradiated cells, it reached approximately 90%, which significantly exceeded the viability levels observed after single-compound treatments. These findings suggest a potential additive effect of CBG and EAA in enhancing melanocyte viability, particularly under oxidative stress induced by UVA irradiation. However, only one concentration combination was tested in this study, which limits broad conclusions regarding interactions between the compounds used.

**Figure 3:**
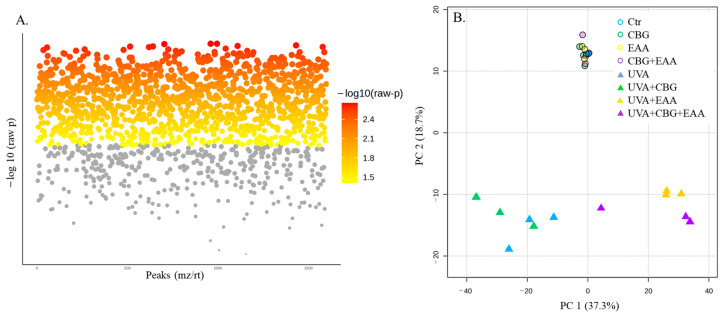
The results of non-parametric Kruskal–Wallis Test (false discovery rate (FDR) < 5%) (**A**) and principal component analysis (PCA) (**B**) performed based on protein expression found in UVA (18 J/cm^2^) irradiated skin melanocytes following 24 h incubation with cannabigerol (CBG, 1 µM), 3-O-ethyl ascorbic acid (EAA, 150 µM), and both compounds used together (CBG + EAA).

**Figure 4:**
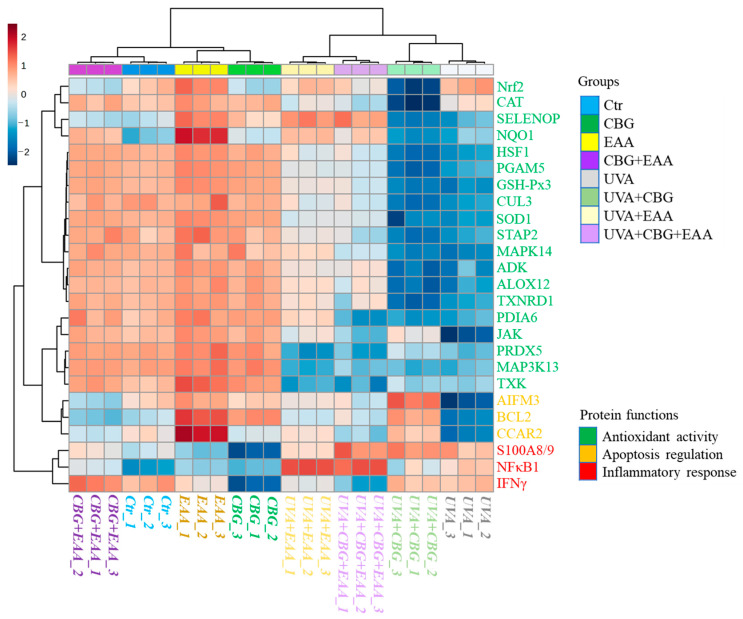
Heatmap and clustering of the top 25 differentially abundant proteins (with *p* value lower than 0.003) identified in UVA (18 J/cm^2^) irradiated skin melanocytes following 24 h incubation with cannabigerol (CBG, 1 µM), 3-O-ethyl ascorbic acid (EAA, 150 µM), and both compounds used together (CBG + EAA). The results of the mass spectrometry data analysis conducted using Proteome Discoverer 2.0 (Thermo Fisher Scientific, Bremen, Germany) are presented in Supplementary File S4. Abbreviations: ADK, adenosine kinase; AIF, apoptosis-inducing factor; ALOX12, arachidonate 12-lipoxygenase; BCL2, B-cell lymphoma 2; CAT, catalase; CCAR, cell cycle and apoptosis regulator; CUL, cullin; GSH-Px, glutathione peroxidase; HSF, heat shock factor; IFN, interferon; JAK, Janus kinase; MAPK, mitogen-activated protein kinase; NFκB, nuclear factor κB; NQO, NAD(P)H: quinone oxidoreductase; Nrf2, nuclear factor erythroid 2-related factor 2; PDI, protein disulfide isomerase; PGAM, phosphoglycerate mutase family member; PRDX, peroxiredoxin; S100A8/9, calprotectin; SELENOP, selenoprotein; SOD, superoxide dismutase; STAP, signal-transducing adaptor protein; TXK, tyrosine-protein kinase; TXNRD1, thioredoxin reductase 1.

Proteomic analysis allowed the identification and semi-quantification of 1599 proteins, among which 1248 were found with statistically significantly changed expression (Figure 3A, Supplementary File S3). Such a number of modified molecules led to a clear separation of samples into melanocytes not exposed to UVA and those exposed to this radiation (Figure 3B). Moreover, non-irradiated cells, regardless of the type of treatment with protectors (CBG, EAA, CBG + EAA), formed one cluster with the Ctr group without internal differentiation, while UVA and UVA + CBG groups were concentrated on the left side of the plot and UVA + EAA and UVA + CBG + EAA groups were shifted to the right. The top 25 proteins with the most strongly differentially abundant expression in experimental melanocytes included mainly proteins involved in cell protection/antioxidant response, as well as pro-inflammatory and proapoptotic signalization (Figure 4). The largest group of proteins downregulated by UVA was antioxidant molecules, but similar changes were also observed in the UVA + CBG group. Moreover, UVA irradiation decreased expression of pro-apoptotic proteins and increased pro-inflammatory factors, which was not observed at such a strong level in the case of UVA-irradiated melanocytes treated with CBG and/or EAA.

**Figure 5:**
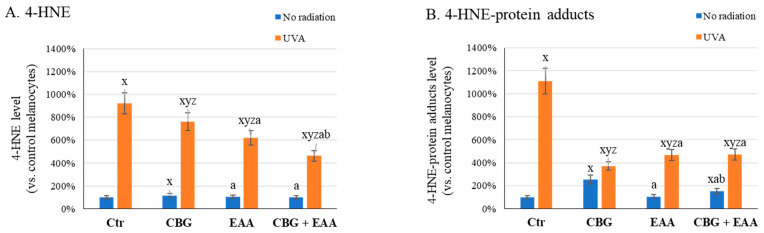
Level of 4-hydroxynonenal (4-HNE) (**A**) and 4-HNE-protein adducts (**B**) in UVA (18 J/cm^2^) irradiated skin melanocytes following 24 h incubation with cannabigerol (CBG, 1 µM), 3-O-ethyl ascorbic acid (EAA, 150 µM), and both compounds used together (CBG + EAA). Mean values ± SD are presented with statistically significant differences comparing to: x—control cells; y—UVA irradiated cells; z—cells treated with the same concentration of CBG or EAA, but not irradiated; a—non irradiated or UVA irradiated cells treated only with CBG (1 µM); b—non irradiated or UVA irradiated cells treated only with EAA (150 µM).

The analysis of oxidative stress markers revealed that the level of 4-HNE significantly increased in melanocytes following UVA irradiation, reaching approximately 900% compared to the non-irradiated control (Figure 5A). Treatment with CBG slightly reduced this increase to around 750%, while EAA exhibited a more pronounced effect, lowering the 4-HNE level to about 600%. The combined application of both compounds (CBG + EAA) resulted in the strongest reduction, with 4-HNE levels dropping below 500%, indicating an additive cytoprotective effect against UVA-induced lipid peroxidation. Importantly, in non-irradiated cells, the levels of 4-HNE remained comparable across all tested conditions and did not significantly differ from the control. In the case of 4-HNE-protein adducts (Figure 5B), UVA exposure led to a marked increase of up to 1100% compared to the non-irradiated control. Treatment with CBG alone considerably reduced this level in irradiated cells to below 400%, whereas EAA lowered it to slightly above 400%. Interestingly, the combined treatment did not enhance this effect further, with 4-HNE-protein adduct levels remaining similar to those observed after EAA treatment alone. In non-irradiated cells, CBG caused an increase in adduct formation to over 200%, while EAA-treated and control cells remained around 100%. Combined treatment in non-irradiated conditions led to a slight elevation (approx. 150%) compared to Ctr, though still markedly lower than CBG alone. The proteins modified by 4-HNE also differed between the studied groups (Table 1). In non-irradiated cells, modified by 4-HNE was a limited number of proteins, and their profiles remained largely unchanged upon treatment with CBG and/or EAA. These included serine/threonine-protein phosphatase 2A, histone acetyltransferase, NADH-cytochrome reductase, and p62 (SQSTM1), which were detected across all non-irradiated groups. Upon UVA exposure, the number and diversity of 4-HNE-modified proteins increased markedly. In untreated irradiated cells, unique adducts were identified on signaling and regulatory proteins such as mitogen-activated protein kinase 10 (MAPK10), ribosomal protein S6 kinase, AP-2 complex subunit, and adenosine kinase, which were not detected in non-irradiated conditions. Treatment with CBG and/or EAA reduced the presence of several UVA-induced adducts, particularly in the combined treatment group, which showed the lowest number of uniquely modified proteins. Notably, key redox-sensitive proteins such as Keap1, IκB kinase, and cullin-3 were detected as targets of 4-HNE adducts formation in irradiated cells, especially in the absence of antioxidant treatment.

These findings suggest that CBG and EAA, particularly when used together, may prevent oxidative modifications of proteins involved in redox regulation, inflammation, and cell signaling, thus contributing to their protective effects observed in melanocytes under UVA-induced oxidative stress.

## 4. Discussion

The proper functioning of the human body is significantly dependent on the condition and functionality of the skin and its cells. This also applies to the body’s protective role against environmental factors, including highly penetrating UVA radiation, which increases the generation of ROS in skin cells, including melanocytes, which, as pigment cells, are also responsible for protecting DNA in other epidermal cells, e.g., keratinocytes [27]. However, continuous exposure of these cells to UVA radiation can alter their metabolism, including redox balance and inflammation, thereby disrupting their functionality and thus weakening the skin’s defenses against harmful environmental factors, which may ultimately promote the development of skin diseases, including cancer [28]. The effects of UVA radiation on skin cells have been studied for many years using various approaches, focusing on targeted changes in the levels of various compounds, including proteins essential for proper cell function, and on the other hand, analyzing modifications in the activity of different enzymes and, consequently, their metabolic effects. The proteomic approach used in this study has the additional advantage of simultaneously assessing changes in protein levels (both enzymatic and signaling) and analyzing changes in their structure [16,29,30]. To date, proteomics has enabled the characterization of numerous pro-inflammatory and pro-apoptotic signaling pathways, as well as pro-/antioxidant cellular responses induced by UVA radiation in skin cells, including the identification of proteins involved in energy management and influencing gene transcription or translation. [14,17,31,32,33]. Consequently, there is a continuing need to support the skin’s natural protective barrier with melanocyte-protective compounds, whose action may be based on preventing changes in the proteomic and metabolic profile of these cells. Considering the varying effectiveness of compounds with proven antioxidant properties on keratinocytes and skin fibroblasts, but with different solubility in a lipophilic-hydrophilic environment [13,14,16,17], the effectiveness of the fat-soluble phytocannabinoid CBG and the water-soluble ascorbic acid derivative EAA on the proteome of UVA-exposed melanocytes was analyzed.

Results obtained in this study indicate that neither CBG nor EAA used separately or together in experimental concentrations have any cytotoxic effect on unirradiated melanocytes, which was previously indicated also in the skin keratinocytes cultured in vitro, where CBG + EAA did not induce any statistically significant changes in the proteome of unirradiated cells [17]. In the case of UVA-irradiated keratinocytes, only CBG showed a protective effect in the context of changes in the proteomic profile [17], while in melanocytes, EAA to a greater extent reduced the changes induced by UVA radiation. Regardless of these effects, CBG more effectively reduces the level of 4-HNE-protein adducts in both keratinocytes [17] and melanocytes after their exposure to UVA radiation. Instead, EAA strongly increases melanocyte viability, which might be the result of EAA-dependent indirect activation of tyrosinase [34], an enzyme that catalyzes the first steps of melanin biosynthesis, which actually drives the metabolism of melanocytes and also causes their increased proliferation [34]. As a result, following cells’ exposure to UVA, EAA treatment of melanocytes (separately, as well as together with CBG) protects cells against the harmful effects of UVA radiation. Moreover, differences in CBG and EAA effects may result from many reasons: on the one hand, they are compounds with different lipophilicity [19], so their target site of action will be different; on the other hand, EAA is primarily a free radical scavenger [35], while CBG may also act by interacting with the endocannabinoid system [36].

### 4.1. Antioxidant Effect of CBG and EAA

Both compounds used are known as strong antioxidants that can directly reduce the level of ROS [37,38], as well as affect the cellular antioxidant system by significantly stimulating its components [39,40]. This study also confirmed that the effects of CBG and EAA on melanocytes are clearly associated with changes in the level of proteins involved in cellular antioxidant defense, particularly after cell exposure to UVA radiation. As a result, there is a reduction in the expression of antioxidant enzymes such as NAD(P)H: quinone oxidoreductase 1 (NQO1), glutathione peroxidase (GSH-PX), superoxide dismutase (SOD), thioredoxin reductase (TXNRD), or peroxiredoxin (PRDX). The decrease in the levels of these enzymes may lead to a reduction in their biological effectiveness and impaired antioxidant function of melanocytes. As a result, the effectiveness of the antioxidant system in UVA-irradiated melanocytes is reduced and accompanied by an increase in ROS generation. This can lead to oxidative modifications of molecules important for cell function, including proteins, lipids, and DNA, which may promote neoplastic transformation of melanocytes, including the development of melanoma [41,42]. Interestingly, CBG used alone does not influence the described UVA-induced changes and therefore potentially has no effect on skin protection against UVA-induced melanoma development, whereas EAA, both used alone and in combination with CBG, reverses all UVA-induced changes in antioxidant protein expression. Similar characteristics of CBG and EAA have been previously observed in UVA-irradiated keratinocytes, where additive effects were demonstrated in the antioxidant effects of EAA and the anti-inflammatory effects of CBG [17].

Additionally, UVA radiation induces the expression of the transcription factor Nrf2 in melanocytes, which leads to the expression of cytoprotective, pro-proliferative, and drug-resistant molecules [43]. Consequently, high Nrf2 overexpression combined with reduced levels of other antioxidant proteins observed following UVA irradiation, may lead to uncontrolled proliferation of cells with oxidative damage, including DNA modifications [44,45]. Independently, UVA radiation induces lipid oxidative metabolism, resulting in increased generation of lipid peroxidation products, including 4-HNE [46]. However, 4-HNE is a highly reactive molecule that can create adducts with proteins, thus influencing their structure, conformation, and activity [12]. Moreover, in melanocytes irradiated with UVA, not only do the levels of 4-HNE and its protein adducts increase, but also significant changes in the profile of proteins modified by 4-HNE are observed. This applies, among others, to the cytosolic Nrf2 inhibitor—protein Keap1, and consequently, its conformational change enables the dissociation of free and active Nrf2 [47]. This change occurs through the oxidation of thiol groups in cysteine residues, which occurs as a result of Keap1’s interaction with 4-HNE [48]. UVA radiation favors 4-HNE-Keap1 adducts formation, which additionally promotes the activation of the Nrf2 [48]. Simultaneously, UVA also induces 4-HNE adducts formation with dipeptidyl peptidase (DPP3), the enzyme that, through interaction with Keap1, increases the release of Nrf2 from the Nrf2-Keap complex [47]. Melanocytes treated with CBG following UVA irradiation significantly reduce the level of Nrf2; however, this effect is not observed after cells are treated with EAA, and in the case of co-treatment, the effect on Nrf2 expression level is intermediate between the effects induced by single compounds. Moreover, CBG and/or EAA, by reducing oxidative stress, also reduce the level of 4-HNE and 4-HNE protein adducts; however, they are unable to eliminate 4-HNE-Keap1 adducts from UVA-irradiated melanocytes. On the other hand, CBG and/or EAA treatment promotes 4-HNE-cullin-3 adducts formation following melanocyte exposure to UVA radiation, which was not observed in cells only irradiated. Cullin-3 is responsible for Nrf2 ubiquitination and marking for degradation [49]; however, there are no data on the effect of 4-HNE adduct formation on the activity of this protein.

Another important factor influencing the activation of Nrf2 is the activity of protein kinases. Nrf2 phosphorylation, depending on the position, may affect the transcriptional activity of this factor in skin cells in various ways, on the one hand, accelerating its translocation to the cell nucleus, or in the case of other phosphorylation sites, causing its removal from the nucleus or preventing its attachment to DNA [50]. As indicated in this study, MAPK10 is one of the main kinases whose activity leads to significant Nrf2 activation [51]. Moreover, following melanocyte irradiation with UVA, MAPK10 is modified by 4-HNE, which leads to the stronger stimulation of this kinase’s phosphorylating activity [52]. As a result, Nrf2 transcriptional activity after UVA radiation is additionally induced. CBG and EAA protective factors used in the experiment fully reverse the modification of MAPK10 by 4-HNE, thereby restoring the physiological activity of this kinase.

On the other hand, UVA also influences antioxidant defense gene expression by the stimulation of histone acetyltransferase activity, thereby facilitating chromatin remodeling and enhancing DNA damage repair mechanisms [53]. Histone acetyltransferase is an enzyme that transfers an acetyl group from acetyl-CoA to lysine residues on histone tails, neutralizing their positive charge. As a result, chromatin structure is relaxed, allowing transcriptional machinery access to DNA, thus regulating DNA repair, as well as cell proliferation and differentiation [54]. There is no literature data on the effect of 4-HNE adducts formation with histone acetyltransferase; however, in connection with the above, it is possible that the prevention of this adducts formation in UVA irradiated cells (also in UVA irradiated cells treated with CBG) additionally promotes transcriptional activity in skin cells under stress conditions.

The presented results indicate how CBG and EAA, by supporting the antioxidant system and partially silencing the Nrf2 factor, promote the regeneration of melanocytes after their exposure to UVA radiation, while simultaneously preventing the overexpression of cytoprotective proteins characteristic of cancer cells [55].

### 4.2. Anti-Inflammatory Effect of CBG and EAA

As a consequence of the changes caused by UVA in melanocytes at the level of redox balance, the level of pro-inflammatory signaling mediators is also increased [56], which may additionally increase melanin production, even leading to hyperpigmentation [57]. Consequently, the results of this study indicate UVA-induced increase in pro-inflammatory proteins, such as NFκB1, IFNγ, and S100A8/9, which is consistent with the literature data [58,59]. Moreover, CBG treatment after UVA radiation partially reduces the levels of these proteins, except NFκB1 in the case of melanocytes treated with EAA or CBG + EAA. The literature data show that EAA has the ability to reduce the activity of the NFκB-dependent pathway; however, there is no data showing the effect of EAA on the NFκB1 (p105) subunit, the high level of which is induced by EAA, indicating the non-canonical pathway of the NFκB activation. This pathway of NFκB activation in melanocytes, as well as melanoma cells, has been described as a factor in the stimulation of the response to inflammation, melanin production, and enhancing skin cell viability [60,61].

Moreover, UVA radiation induces 4-HNE adduct formation with IκB kinase (IKK) in melanocytes. IKK, in turn, phosphorylates IκB, which leads to its degradation and release/nuclear transport of active NFκB, resulting in pro-inflammatory messengers biosynthesis. It is known that 4-HNE binding to the kinases responsible for IκB phosphorylation results in inhibition of IκB and favors NFκB activation [12]. EAA, as well as CBG, accompanied by EAA, prevents the formation of 4-HNE-IKK adducts, which can be considered an additional mechanism limiting pro-inflammatory signaling in melanocytes exposed to UVA radiation.

### 4.3. Effect of CBG and EAA on Apoptosis

UVA radiation is a known factor that reduces the viability of skin cells [19]; however, in the case of melanocytes, which are evolutionarily prepared to respond to this radiation through melanin synthesis, small doses of radiation can stimulate the metabolic activity of melanocytes and even promote their proliferation [62]. This pro-proliferative effect of UVA on melanocytes, along with their protection against apoptosis, observed in this study as a reduction in proapoptotic proteins, such as AIFM3, BCL2, and CCAR2, may even promote melanoma development. CBG and EAA used following cell irradiation partially prevent this strong decrease in pro-apoptotic protein expression, thus enabling the neutralization of cells through programmed cell death in cases where they are severely damaged by UVA. Moreover, this effect is most strongly observed when melanocytes are co-treated with CBG and EAA, which suggests cooperation of compounds in this area of action.

Furthermore, it has also been suggested that 4-HNE protein adduct formation often promotes intercellular pro-apoptotic signalization [48]. In the case of melanocytes, UVA radiation does not induce 4-HNE adduct formation with p62, while both CBG and EAA lead to the formation of adducts of this protein with 4-HNE in both irradiated and non-irradiated cells. However, the multifunctional protein p62 links pro-inflammatory signaling with autophagy and apoptosis [63], and its accumulation, caused by impaired decomposition, induces cell death; therefore, marking p62 by 4-HNE, which disrupts its degradation, may direct melanocytes with UVA-induced excess lipid peroxidation product levels for apoptosis. A similar situation is observed in the case of protein kinase C (PKC), which is modified by 4-HNE mainly following cell treatment with CBG and/or EAA, but not only after UVA irradiation. 4-HNE adducts with PKC activate this enzyme and promote the natural elimination of damaged skin cells, thus eliminating the risk of their proliferation and cancer formation [64]. Therefore, it can be suggested that CBG and EAA can support healthy skin function by directing melanocytes with modified proteins to the apoptosis pathway and will not allow UVA-mutated cells to proliferate.

## 5. Conclusions

The proteomic results obtained in this study indicate that CBG and EAA, when used together, partially protect melanocytes from the effects of UVA radiation. Furthermore, the results suggest a partial protective effect of EAA on the antioxidant system and CBG’s effect on pro-inflammatory and pro-apoptotic signaling pathways. However, this study is limited by the nature of proteomic studies, which allow only semi-quantitative analyses, yielding only descriptive results with an attempt to analyze them at the functional level. This applies to both changes in protein expression and intracellular signaling based on protein modification by lipid peroxidation products. In summary, it is suggested that CBG and EAA may provide partial protection to melanocytes from the effects of UVA-induced oxidative stress and possibly also partially protect them from carcinogenesis.

## Figures and Tables

**Table 1:** List of protein adducts with 4-hydroxynonenal (4-HNE) in UVA (18 J/cm^2^) irradiated skin melanocytes following 24 h incubation with cannabigerol (CBG, 1 µM), 3-O-ethyl ascorbic acid (EAA, 150 µM), and both compounds used together (CBG + EAA). Fields marked with “+” indicate the presence of modifications in individual samples.

4-HNE-Modified Protein	Melanocytes							
	Non-Irradiated				UVA			
	−	CBG	EAA	CBG + EAA	+	CBG	EAA	CBG + EAA
serine/threonine-protein phosphatase 2A (E9PMD7)	+	+	+	+				
histone acetyltransferase (Q92830)	+	+	+	+			+	+
NADH-cytochrome reductase (P00387)	+	+	+	+		+	+	+
p62 (SQSTM1)	+	+	+	+		+	+	+
protein kinase C (P17252)		+		+		+	+	+
cullin-3 (Q13618)		+		+		+	+	+
dipeptidyl peptidase 3 (Q9NY33)		+			+	+	+	+
Keap1 (Q14145)		+			+	+	+	+
serpin A12 (Q8IW75)					+	+		
IκB kinase (O15111)					+	+		
adenosine kinase (P55263)					+	+		
mitogen-activated protein kinase 10 (P53779)					+			
ribosomal protein S6 kinase (P23443)					+			
AP-2 complex (Q92481)					+			

## Data Availability

The authors confirm that the data supporting the findings of this study are available within the Supplementary Materials. The mass spectrometry proteomics data have been deposited in the ProteomeXchange Consortium via the PRIDE [65] partner repository with the dataset identifier PXD074276.

## Supplementary Materials

The following supporting information can be downloaded at: https://www.mdpi.com/article/10.3390/cells15110965/s1. Supplementary File S1. The details of the method parameters for proteins identification and quantification used for melanocytes analyses following their UVA (18 J/cm^2^) irradiation and 24 h incubation with cannabigerol (CBG, 1 µM), 3-O-ethyl ascorbic acid (EAA, 150 µM) and both compounds used together (CBG + EAA). Supplementary File S2. The results of data normalization by the median of the protein intensities and log-transformation using open-source software MetaboAnalyst 6.0 (http://www.metaboanalyst.ca; accessed on 11 September 2025). Supplementary File S3. IDs of the proteins that were identified and semi-quantified in UVA (18 J/cm^2^) irradiated skin melanocytes following 24 h incubation with cannabigerol (CBG, 1 µM), 3-O-ethyl ascorbic acid (EAA, 150 µM) and both compounds used together (CBG + EAA). Data obtained using high-performance liquid chromatography system Ultimate 3000 (Dionex, Idstein, Germany) coupled Q Exactive HF mass spectrometer (Thermo Fisher Scientific, Bremen, Germany). Raw data were analyzed by the Proteome Discoverer 2.0 (Thermo Fisher Scientific, Seattle, WA, USA) against the UniProtKB-SwissProt database (taxonomy: Homo sapiens, release 2025-04). Supplementary File S4. The results of the mass spectrometry data analysis of the top 25 differentially abundant proteins (with *p* value lower than 0.003) identified in UVA (18 J/cm^2^) irradiated skin melanocytes following 24 h incubation with cannabigerol (CBG, 1 µM), 3-O-ethyl ascorbic acid (EAA, 150 µM) and both compounds used together (CBG + EAA). Analysis conducted using Proteome Discoverer 2.0 (Thermo Fisher Scientific, Bremen, Germany) against the UniProtKB-SwissProt database (taxonomy: Homo sapiens, release 2025-04).

## Abbreviations

The following abbreviations are used in this manuscript:

^1^O_2_	singlet oxygen
4-HNE	4-hydroxynonenal
8-oxoGua	8-oxo-7,8-dihydroguanine
ADK	adenosine kinase
AIF	apoptosis-inducing factor
ALOX12	arachidonate 12-lipoxygenase
ANOVA	univariate analysis one-way
ATCC	American Type Culture Collection
BCL2	B-cell lymphoma 2
CAT	catalase
CBG	cannabigerol
CCAR	cell cycle and apoptosis regulator
COX	cyclooxygenase
CPD	cyclobutanepyrimidine dimer
CUL	cullin
DCBM	dermal cell basal medium
DPP	dipeptidyl peptidase
EAA	3-*O*-ethylascorbic acid
FDR	false discovery rate
GSH-Px	glutathione peroxidase
H_2_O_2_	hydrogen peroxide
HSF	heat shock factor
IFN	interferon
IKK	IκB kinase
JAK	Janus kinase
LC/MS	liquid chromatography-mass spectrometry
LOX	lipoxygenase
LSD	least significant differences
MAPK	mitogen-activated protein kinase
MDA	malondialdehyde
MTT	3-(4,5-dimethylthiazol-2-yl)-2,5-diphenyltetrazolium bromide
NFκB	nuclear factor κB
NO	nitric oxide
NQO	NAD(P)H: quinone oxidoreductase
Nrf2	nuclear factor erythroid 2-related factor 2
O_2_^−^	superoxide anion
PANTHER	Protein Analysis Through Evolutionary Relationships Classification System
PBS	phosphate-buffered saline
PCA	principal component analysis
PDI	protein disulfide isomerase
PGAM	phosphoglycerate mutase family member
PRDX	peroxiredoxin
ROS	reactive oxygen species
S100A8/9	calprotectin
SD	standard deviation
SDS-PAGE	sodium dodecyl sulfate-polyacrylamide gel electrophoresis
SELENOP	selenoprotein
SOD	superoxide dismutase
STAP	signal-transducing adaptor protein
TXK	tyrosine-protein kinase
TXNRD1	thioredoxin reductase 1
UVA	ultraviolet A
