# Supplementary material for: Proteomic Analysis of the Metabolic Response of UVA-Exposed Melanocytes Following Co-Treatment with Cannabigerol and 3-O-Ethylascorbic Acid
Source: Cells. 2026 May 23;15(11):965. doi: 10.3390/cells15110965 (PMC13256526; doi:10.3390/cells15110965)
Supplement: Supplementary file 1 [file cells-15-00965-s001.zip › S1.pdf]

Supplementary file S1. The details of the method parameters for proteins identification and quantification used for melanocytes analyses following their UVA (18 J/cm^2) irradiation and 24h incubation with cannabigerol (CBG, 1 µM), 3-O-ethyl ascorbic acid (EAA, 150 µM) and both compounds used together (CBG + EAA).

A. ***Method of UHPLC Dionex UltiMate3000 RSLCnano System*** (Dionex, Idstein, Germany) with a 150 mm x 75 µm PepMap RSLC capillary analytical C18 column (Dionex, LC Packings)

Instrument Setup

UV.TimeConstant	0.12 [s]
UV.Data_Collection_Rate	10.0 [Hz]
Sampler.Temperature.Nominal	5.0 [°C]
PumpModule.LoadingPump.Pressure.LowerLimit	0 [bar]
PumpModule.LoadingPump.Pressure.UpperLimit	500 [bar]
PumpModule.NC_Pump.Pressure.LowerLimit	2 [bar]
PumpModule.NC_Pump.Pressure.UpperLimit	800 [bar]
PumpModule.NC_Pump.MaximumFlowRampUp/Down	0.300 [µl/min²]
ColumnOven.Temperature.Nominal	40.0 [°C]
UV.UV_VIS_1.Wavelength	214 [nm]

Time 0.000

Inject Preparation

Sampler.Inject

Start Run

Duration	60.000 [min]
PumpModule.LoadingPump.Flow.Nominal	5.000 [µl/min]
PumpModule.NC_Pump.Flow.Nominal	0.300 [µl/min]
PumpModule.NC_Pump.%B.Value	4.0 [%]

Time 3.000

PumpModule.NC_Pump.Flow.Nominal	0.300 [µl/min]
PumpModule.NC_Pump.%B.Value	4.0 [%]

Time 40.000

PumpModule.NC_Pump.Flow.Nominal	0.300 [µl/min]
PumpModule.NC_Pump.%B.Value	55.0 [%]

Time 44.000

PumpModule.NC_Pump.Flow.Nominal	0.300 [µl/min]
PumpModule.NC_Pump.%B.Value	90.0 [%]

Time 50.000

PumpModule.NC_Pump.Flow.Nominal	0.300 [µl/min]
PumpModule.NC_Pump.%B.Value	4.0 [%]

Time 60.000

PumpModule.LoadingPump.Flow.Nominal	5.000 [µl/min]
PumpModule.NC_Pump.Flow.Nominal	0.300 [µl/min]
PumpModule.NC_Pump.%B.Value	4.0 [%]

Stop Run

End

B. ***Method of Q Exactive HF*** with an electrospray ionization source (ESI) (Thermo Fisher Scientific, Bremen, Germany)

Overall method settings

**Global Settings**	
Chrom. peak width (FWHM)	15 s
**Time**	
Method duration	60.00 min

Full MS/dd-MS² (TopN)

**General**	
Runtime	0 to 60 min
Polarity	positive
In-source CID	0.0 eV
Default charge state	2
**Full MS**	
Microscans	1
Resolution	120,000
AGC target	3e6
Maximum IT	100 ms
Number of scan ranges	1
Scan range	200 to 2000 m/z
Spectrum data type	Profile
**dd-MS²/dd-SIM**	
Microscans	1
Resolution	30,000
AGC target	1e5
Maximum IT	50 ms
Loop count	5
MSX count	1
TopN	5
Isolation window	4.0 m/z
Isolation offset	0.0 m/z
Scan range	200 to 2000 m/z
Spectrum data type	Profile
**dd Settings**	
Minimum AGC target	1.00e3
Intensity threshold	2.0e4
Multiple charge states	all
Peptide match	preferred
Exclude isotopes	on
Dynamic exclusion	10.0 s

C. ***<u>Method of data processing using Proteome Discoverer 2.0</u>*** (Thermo Fisher Scientific, Bremen, Germany)(searched against the UniProtKB - SwissProt database (taxonomy: Homo sapiens, release 2025-04))

<u>Workflow of processing step</u>

Processing node 1: Spectrum Selector

General Settings:
- Precursor Selection: Use MS1 Precursor
- Use New Precursor Reevaluation: True
- Use Isotope Pattern in Precursor Reevaluation: True

Spectrum Properties Filter:
- Lower RT Limit: 0
- Upper RT Limit: 0
- First Scan: 0
- Last Scan: 0
- Lowest Charge State: 0
- Highest Charge State: 0
- Min. Precursor Mass: 350 Da
- Max. Precursor Mass: 5000 Da
- Total Intensity Threshold: 0
- Minimum Peak Count: 1

Scan Event Filters:
- MS Order: Is Not MS1
- Min. Collision Energy: 0
- Max. Collision Energy: 1000
- Scan Type: Is Full

Peak Filters:
- S/N Threshold (FT-only): 1.5

Replacements for Unrecognized Properties:
- Unrecognized Charge Replacements: Automatic
- Unrecognized Mass Analyzer Replacements: ITMS
- Unrecognized MS Order Replacements: MS2
- Unrecognized Activation Type Replacements: CID
- Unrecognized Polarity Replacements: +
- Unrecognized MS Res@200 Replacements: 60000
- Unrecognized MSn Res@200 Replacements: 30000

Precursor Pattern Extraction:
- Precursor Clipping Range Before: 2.5 Da
- Precursor Clipping Range After: 5.5 Da

Processing node 2: MS Amanda

Input Data:
- Protein Database: Homo sapiens, release 2018-04
- Enzyme Name: Trypsin
- Missed Cleavages: 2
- MS1 tolerance: 5 ppm
- MS2 tolerance: 0.02 Da

Dynamic Modifications:

Dynamic Modification: Carbamidomethyl
    Substitution= "H(3) C(2) N O" PositionType="Any" />

Dynamic Modification: Carbamyl
    Substitution= "H C N O" PositionType="Any" />

Dynamic Modification: 4-HNE
    Substitution= "H(16) C(9) O (2)" PositionType="Any" />

Additional Settings:
- Max No. of same modifs: 3

- Max No. of dynamic modifs:	4
- Ion Settings:	b,y
- Max. Rank:	5
- Max number of same neutral losses (H2O, NH3):	1
- No. of considered NLs (modifications):	2
- Perform deisotoping:	True
- Use monoisotopic mass:	True

Processing node 3: Percolator
Input Data:

- Maximum Delta Cn:	0.05

Decoy Database Search:

- Target FDR (Strict):	0.01
- Target FDR (Relaxed):	0.05
- Validation based on:	q-Value

Processing node 5: Event Detector
General Settings:

- Mass Precision:	2 ppm
- S/N Threshold:	1

Processing node 6: Precursor Ions Area Detector
No parameters

Workflow of consensus step
Processing node 1: PSM Grouper
Peptide Group Modifications:

- Site Probability Threshold:	75

Display Options:

- Modification Sites Shown:	Best Position

Processing node 2: Peptide Validator
General Validation Settings:

- Validation Mode:	Automatic (Control peptide level error rate if possible)
- Target FDR (Strict) for PSMs:	0.01
- Target FDR (Relaxed) for PSMs:	0.05
- Target FDR (Strict) for Peptides:	0.01
- Target FDR (Relaxed) for Peptides:	0.05

Specific Validator Settings:

- Validation Based on:	q-Value

- Use Concatenated FDR Calculation for PSM Level FDR Calculation Based on Score: False
- Reset Confidences for Nodes without Decoy Search (Fixed score thresholds): False

Processing node 3: Peptide and Protein Filter
Peptide Filters:

- Peptide Confidence At Least:	Medium
- Keep Lower Confident PSMs:	False
- Minimum Peptide Length:	6
- Remove Peptides Without Protein Reference:	False

Protein Filters:

- Minimum Number of Peptide Sequences:	1
- Count Only Rank 1 Peptides:	False
- Count Peptides Only for Top Scored Protein:	False

Processing node 4: Protein Scorer
No parameters

Processing node 5: Protein Grouping

Protein Grouping:
- Apply strict parsimony principle: True

Processing node 6: Protein FDR Validator
Confidence Thresholds:
- Target FDR (Strict): 0.01
- Target FDR (Relaxed): 0.05

Processing node 7: Peptide and Protein Quantifier
Ratio Calculation:
- Minimum Quan Value Threshold: 0.0001
- Replace Missing Quan Values With Minimum Intensity: False
- Reject All Quan Values If Not All Quan Channels Are Present: False
- Maximum Allowed Fold Change: 100
- Use Ratios Above Maximum Allowed Fold Change for Quantification: False
- Create Separate Quan Columns: False
Ratio Calculation for Precursor Quan:
- Use Single-Peak Quan Channels: False
Ratio Calculation for Reporter Quan:
- Apply Quan Value Corrections: True
- Co-Isolation Threshold: 100
Protein Quantification:
- Use Only Unique Peptides: True
- Consider Proteins Groups for Peptide Uniqueness: True
- Top N Peptides Used for Area Calculation: 3
Normalization:
- Experimental Bias Correction: None
- Minimum Ratio Count for Median Normalization: 20
- Manual Normalization Factor: 1
Display Options:
- Show the Raw Quan Values: True
- Show Standard Error: True
- Show Ratio Variabilities: False
- Show Ungrouped Ratios: False
- Show Ratio Counts: False
Quan Ratio Distributions:
- 1st Fold Change Threshold: 2
- 2nd Fold Change Threshold: 4
- 3rd Fold Change Threshold: 6
- 4th Fold Change Threshold: 8
- 5th Fold Change Threshold: 10
