# Supplementary material for: Proteomic Analysis of the Metabolic Response of UVA-Exposed Melanocytes Following Co-Treatment with Cannabigerol and 3-O-Ethylascorbic Acid
Source: Cells. 2026 May 23;15(11):965. doi: 10.3390/cells15110965 (PMC13256526; doi:10.3390/cells15110965)

Supplementary file S2. The results of data normalization by the median of the protein intensities and log-transforation using open-source software MetaboAnalyst 6.0 (http://www.metaboanalyst.ca; accessed on 11 September 2025).

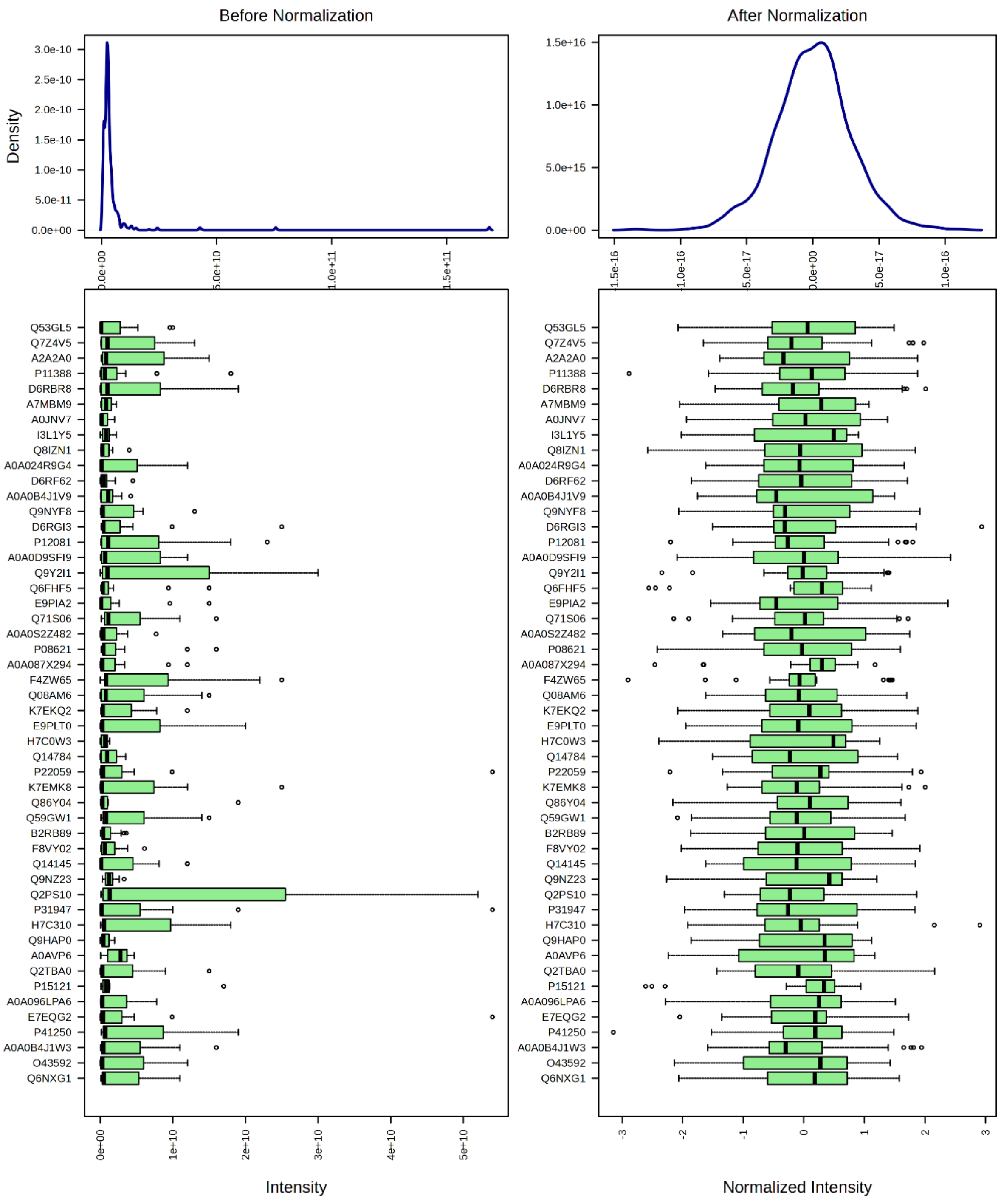

Supplement: Supplementary file 1 [file cells-15-00965-s001.zip › S2.pdf]
